# Zaizao Shengxue capsule for anemia: a prospective multicenter real-world study of hemoglobin response and predictive modeling

**DOI:** 10.3389/fphar.2026.1957386

**Published:** 2026-10-19

**Authors:** Fuwei Xing, Jiaxin Wu, Cheng Cheng, Siyu Chen, Zihan Qin, Yuzhuo Wang, Yuqian Zhang, Junzi Tang, Xiao Li

**Affiliations:** Dongzhimen Hospital of Beijing University of Chinese Medicine, Beijing, China

**Keywords:** anemia, machine learning, real-world study, traditional Chinese medicine, Zaizao Shengxue capsule

## Abstract

**Objective:**

The aim in this study was to characterize the 8-week hemoglobin response among patients with anemia receiving Zaizao Shengxue capsule (ZSC) in real-world care and develop and externally evaluate a model for estimating the individual probability of response.

**Methods:**

This prospective multicenter study included 440 patients from 14 centers who received ZSC for 8 weeks. Response was defined as an increase in hemoglobin of at least 20 g/L from baseline at week 8. The development cohort (*n* = 338) was divided into a training cohort (*n* = 238) and an internal validation cohort (*n* = 100). Baseline predictors were selected in the training cohort. Ten candidate models were trained using different algorithms and compared in the internal validation cohort. After model selection and locking, the final model was independently evaluated in 102 patients from four external centers. SHAP and LIME were used for model interpretation.

**Results:**

Overall, 204 of 440 patients (46.4%) achieved an 8-week hemoglobin response. Response rates varied across anemia etiologies: 24.0% in cancer-related anemia (60/250), 74.4% in iron-deficiency anemia (67/90), and 77.0% in anemia of chronic disease (77/100). Anemia etiology, age, and estimated glomerular filtration rate (eGFR) were retained as predictors in the final Extra Trees model. The model achieved an area under the receiver operating characteristic curve (AUC) of 0.821 (95% CI, 0.729–0.902) in internal validation and 0.769 (95% CI, 0.670–0.860) in external validation. SHAP analysis identified anemia etiology as the largest contributor to model predictions, followed by age and eGFR.

**Conclusion:**

Eight-week hemoglobin response varied across anemia etiologies among patients receiving ZSC. A model incorporating anemia etiology, age, and eGFR may support individualized pretreatment estimation of response probability based on routinely available clinical information.

## 1 Introduction

Anemia is a heterogeneous clinical condition arising from nutritional deficiency, chronic inflammation, malignancy, treatment-related marrow suppression, bleeding, and other causes. Patients receiving the same intervention may differ in their hemoglobin response because of anemia etiology, age, renal function, disease burden, and other baseline characteristics. Prediction tools based on routinely available pretreatment information may help clinicians assess the probability of response in individual patients.

Zaizao Shengxue capsule (ZSC) is a commercial Chinese polyherbal preparation used to treat anemia. Its approved indications include aplastic anemia and iron-deficiency anemia (Li et al., 2025a). ZSC contains 21 medicinal ingredients, including Tu Si Zi, Hong Shen, E Jiao, Dang Gui, Huang Qi, Bai Shao, and Shu Di Huang; detailed information on their source species, families, pharmacopeial drug names, medicinal parts, and labeled processing forms is provided in Supplementary Table S2 (Chinese Pharmacopoeia Commission, 2020; Heinrich et al., 2022). Previous clinical studies have reported improvements in hematologic parameters in patients with myelodysplastic syndrome and iron-deficiency anemia (Ding et al., 2020; Wang et al., 2025). A murine study also reported changes in peripheral blood-cell indices and bone-marrow cellularity following ZSC administration (Yang et al., 2025).

Machine learning has been applied to the differential diagnosis and risk prediction of anemia using complete blood counts and other clinical variables (Darshan et al., 2025; Su et al., 2024). Predicting hemoglobin response addresses a different clinical question: whether a patient is likely to achieve a predefined increase in hemoglobin after treatment. For patients receiving ZSC, routinely available baseline information may provide a practical basis for predicting hemoglobin response, although model performance may not generalize across clinical settings (la Roi-Teeuw et al., 2024; Efthimiou et al., 2024). We, therefore, aimed to develop and externally evaluate a model for estimating the probability of an 8-week hemoglobin response among patients receiving ZSC in real-world care. Model explanations were used to describe predictions rather than infer causal treatment effects (Carriero et al., 2025).

## 2 Materials and methods

### 2.1 Study design and participant recruitment

This prospective, multicenter, real-world study was conducted at 14 centers in China from May 2023 to December 2025. Local investigators screened patients in routine clinical practice according to prespecified eligibility criteria. Eligible patients were consecutively approached and enrolled after providing written informed consent. The study was reported in accordance with the TRIPOD + AI reporting guidance (Collins et al., 2024).

### 2.2 Eligibility criteria

Patients were eligible if they: (1) had anemia; (2) were 18–80 years old; (3) agreed to participate in the study; and (4) provided written informed consent. Anemia was defined for this study as a baseline hemoglobin concentration <120 g/L (<12 g/dL) in men or <110 g/L (<11 g/dL) in non-pregnant women.

Patients were excluded at enrollment if they: (1) were participating in another clinical trial; (2) were pregnant, lactating, or planning pregnancy; (3) had participated in another drug trial within the preceding month; (4) had a known allergy or hypersensitivity to ZSC or any of its ingredients; or (5) were using other Chinese medicinal preparations or herbal decoctions intended to improve anemia.

Anemia etiology was classified according to the primary clinical diagnosis recorded by the enrolling physician as cancer-related anemia, iron-deficiency anemia, or anemia of chronic disease. Cancer-related anemia was defined as anemia clinically attributed to active malignancy and/or antineoplastic treatment. Iron-deficiency anemia was defined using the available clinical history, complete blood count findings, and iron-status tests when available. Anemia of chronic disease was defined as anemia clinically attributed to an underlying chronic inflammatory, infectious, autoimmune, renal, or other chronic non-malignant disorder.

### 2.3 Data collection

Study data were recorded in a standardized electronic data capture (EDC) system. Investigators at each center entered data according to the prespecified study protocol. Baseline data included demographic characteristics, anemia-related clinical information, comorbidities, routine laboratory measurements, and histories of anemia treatment and transfusion. Follow-up data from the 8-week treatment period were recorded in the same system.

### 2.4 Treatment and follow-up

The study used an approved, commercially manufactured ZSC product supplied by Anhui Leiyunshang Pharmaceutical Co., Ltd., China [0.32 g per capsule; 90 capsules per bottle; National Medical Products Administration (NMPA) approval No. Z20194065]. Supplementary Table S2 summarizes the source taxa, medicinal parts, and labeled processing forms of the ingredients. The product was from batch 20230504, recorded as batch No. 230504 in the manufacturer’s finished-product inspection report. The manufacturer-issued Certificate of Analysis is provided as a separate Supplementary Material. Patients received five capsules orally three times daily for eight consecutive weeks.

Follow-up assessments were scheduled at weeks 2, 4, 6, and 8. At each visit, investigators recorded clinical status, concomitant medications and treatments, treatment adherence, adverse events, and relevant laboratory measurements in the EDC system. Missed assessments were recorded as missing. Patients without an evaluable week-8 hemoglobin (HGB) measurement were excluded from the prediction-model analysis. Week-8 HGB values were not imputed.

Concomitant treatments were reviewed throughout the 8-week follow-up. Patients receiving additional interventions expected to directly affect HGB were excluded from the prediction-model analysis. These interventions included antitumor therapy, iron supplementation or other anemia-directed therapy, blood transfusion, and erythropoiesis-directed therapy. Counts by treatment category are provided in Supplementary Table S7.

### 2.5 Outcome definition

The primary outcome was hemoglobin response at week 8: patients with an HGB increase of at least 20 g/L (2 g/dL) from baseline were classified as responders, whereas those who did not meet this threshold were classified as non-responders. This threshold was based on published criteria for hematologic treatment-response assessment (Shen and Zhao, 2018). Because the likelihood of achieving this increase may depend on baseline HGB, baseline hematologic variables were considered as candidate predictors.

### 2.6 Candidate predictors and missing-data handling

Thirty-two pretreatment variables were initially reviewed, covering demographics, anemia characteristics, comorbidities, prior treatment, and routine laboratory measurements. The estimated glomerular filtration rate (eGFR) was calculated from serum creatinine, age, and sex using the 2021 CKD-EPI creatinine equation and expressed in mL/min/1.73 m^2^ (Inker et al., 2021). Redundancy among continuous variables was assessed using Pearson and Spearman correlation coefficients (Supplementary Figure S3). Following correlation-based redundancy screening, collinearity was assessed using variance inflation factors (VIFs; Supplementary Table S6). This screening process yielded 24 candidate predictors for feature selection. Missing continuous and categorical values were imputed using the median and mode, respectively. Imputation values were derived exclusively from the training cohort and applied unchanged to the internal and external validation cohorts. Missing-data distributions before imputation are summarized in Supplementary Figure S1.

### 2.7 Dataset construction

Four centers formed the external validation cohort (*n* = 102). The remaining 10 centers formed the development cohort (*n* = 338), which was divided by stratified random sampling into training (*n* = 238) and internal validation (*n* = 100) cohorts. Stratification by the response status preserved the outcome proportion in the development split. External-validation patients were not used for feature selection, model fitting, hyperparameter tuning, or final algorithm selection.

### 2.8 Feature selection and model development

Feature selection was performed in the full training cohort before cross-validation. Five methods were applied: multivariable logistic regression, LASSO using the one-standard-error rule, Boruta, random-forest recursive feature elimination (RF-RFE), and a genetic algorithm (Friedman et al., 2010; Kursa et al., 2010). For voting, the RF-RFE and genetic-algorithm selections were each restricted to the five highest-ranked variables. Predictors selected by at least four of the five methods were retained for model development (Pudjihartono et al., 2022).

Ten algorithms were trained using the selected predictors: decision tree (DT), XGBoost, support vector machine (SVM), artificial neural network (ANN), linear discriminant analysis (LDA), and extremely randomized trees (Extra Trees; ET). The remaining algorithms were Gaussian naive Bayes (GNB), logistic regression (LR), LightGBM, and CatBoost (Pedregosa et al., 2011; Chen and Guestrin, 2016; Ke et al., 2017; Prokhorenkova et al., 2018). Hyperparameters were tuned by stratified five-fold cross-validation in the training cohort after feature selection. Selected settings and mean cross-validated areas under the receiver operating characteristic curve (AUCs) are reported in Supplementary Table S4. Pooled out-of-fold ROC curves are shown in Supplementary Figure S5.

### 2.9 Model evaluation and interpretation

Discrimination was assessed using the AUC, with 95% confidence intervals. Accuracy, sensitivity, specificity, precision, negative predictive value (NPV), and F1 score were calculated at a probability threshold of 0.50. Calibration was assessed using plots, Brier scores, calibration intercepts, and calibration slopes. Decision curve analysis assessed net benefit (Vickers and Holland, 2021). Precision–recall curves were generated for both validation cohorts and summarized using average precision (AP) (Saito and Rehmsmeier, 2015). Pairwise DeLong comparisons in the internal validation cohort were exploratory and were not adjusted for multiple comparisons (Robin et al., 2011).

SHAP was used to describe global and individual contributions to model predictions, and LIME provided a complementary local explanation (Lundberg and Lee, 2017; Ribeiro et al., 2016). These analyses described model predictions and were not interpreted as causal effects. ET was selected based on its internal-validation results, including the highest AUC and AP, together with classification and calibration performance. Thus, the internal validation cohort informed the final algorithm selection. The final ET model was locked after internal validation and then evaluated in the external validation cohort without refitting.

## 3 Results

### 3.1 Study population

Of the 589 enrolled patients, 77 lacked an evaluable week-8 HGB outcome. During follow-up, 82 patients received concomitant interventions considered likely to directly affect HGB; 10 of these patients also lacked an evaluable outcome. Excluding the 77 patients with missing outcomes and an additional 72 patients receiving such interventions left 440 patients in the analysis cohort (Figure 1). The development cohort comprised 338 patients from 10 centers, divided into training (*n* = 238) and internal validation (*n* = 100) cohorts. The external validation cohort comprised 102 patients from four centers; its baseline characteristics are presented in Supplementary Table S1. Overall, 204/440 patients (46.4%) achieved a response. The corresponding counts were 109/238 (45.8%), 46/100 (46.0%), and 49/102 (48.0%) in the training, internal validation, and external validation cohorts, respectively. The response rates were 24.0% in cancer-related anemia (60/250), 74.4% in iron-deficiency anemia (67/90), and 77.0% in anemia of chronic disease (77/100).

**FIGURE 1:**
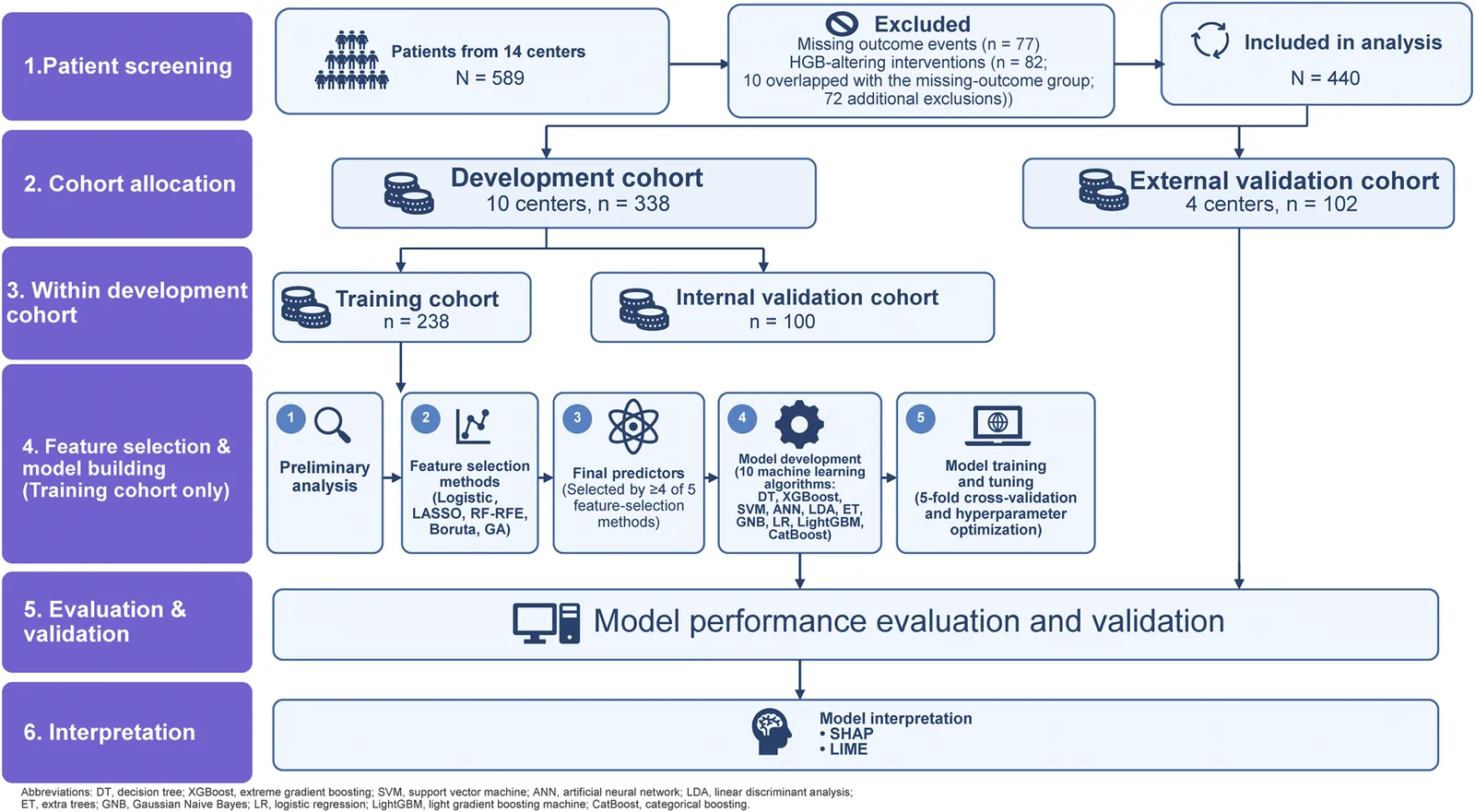
Study flow and cohort allocation.

### 3.2 Training-cohort baseline characteristics

Table 1 summarizes pretreatment characteristics in the training cohort according to the 8-week hemoglobin-response status.

**TABLE 1:** Baseline characteristics of the training cohort stratified by 8-week hemoglobin response.

Variable	Overall (*N* = 238)	Non-response (*N* = 129)	Response (*N* = 109)	*P*
Sex	​	​	​	0.007
Female	164 (68.91%)	79 (61.24%)	85 (77.98%)	​
Male	74 (31.09%)	50 (38.76%)	24 (22.02%)	​
Anemia etiology	​	​	​	<0.001
Cancer-related anemia	135 (56.72%)	103 (79.84%)	32 (29.36%)	​
Iron-deficiency anemia	43 (18.07%)	11 (8.53%)	32 (29.36%)	​
Anemia of chronic disease	60 (25.21%)	15 (11.63%)	45 (41.28%)	​
Anti-anemia treatment history	​	​	​	0.142
No	220 (92.44%)	116 (89.92%)	104 (95.41%)	​
Yes	18 (7.56%)	13 (10.08%)	5 (4.59%)	​
Transfusion history	​	​	​	0.234
No	227 (95.38%)	121 (93.80%)	106 (97.25%)	​
Yes	11 (4.62%)	8 (6.20%)	3 (2.75%)	​
Hypertension	​	​	​	0.001
No	208 (87.39%)	104 (80.62%)	104 (95.41%)	​
Yes	30 (12.61%)	25 (19.38%)	5 (4.59%)	​
Smoking	​	​	​	0.379
No	216 (90.76%)	115 (89.15%)	101 (92.66%)	​
Yes	22 (9.24%)	14 (10.85%)	8 (7.34%)	​
Alcohol consumption	​	​	​	0.070
No	222 (93.28%)	124 (96.12%)	98 (89.91%)	​
Yes	16 (6.72%)	5 (3.88%)	11 (10.09%)	​
Age (years)	63.00 [50.25, 71.00]	68.00 [58.00, 72.00]	54.00 [43.00, 69.00]	<0.001
BMI (kg/m^2^)	22.66 [20.62, 24.65]	22.66 [20.34, 24.25]	22.94 [20.81, 25.39]	0.083
HGB (g/L)	95.50 [86.00, 103.00]	97.00 [87.00, 105.00]	92.00 [85.00, 101.00]	0.006
WBC (×10^9^/L)	5.52 [3.90, 7.81]	4.85 [3.36, 7.20]	6.36 [4.65, 8.63]	<0.001
RBC (×10^12^/L)	3.34 [2.90, 3.68]	3.28 [2.89, 3.68]	3.42 [2.98, 3.67]	0.174
PLT (×10^9^/L)	200.00 [139.75, 268.75]	180.00 [97.00, 251.00]	230.00 [188.00, 277.00]	<0.001
LYMPH (×10^9^/L)	1.06 [0.76, 1.52]	1.04 [0.66, 1.41]	1.20 [0.80, 1.59]	0.066
MONO (×10^9^/L)	0.40 [0.25, 0.58]	0.33 [0.21, 0.55]	0.47 [0.30, 0.60]	0.003
NEUT (×10^9^/L)	3.70 [2.43, 5.69]	3.23 [2.04, 5.06]	4.19 [3.00, 6.30]	<0.001
HCT (%)	29.40 [26.65, 32.00]	30.00 [26.90, 32.50]	28.70 [26.50, 31.40]	0.044
MCV (fL)	89.15 [83.62, 95.05]	92.30 [86.90, 97.50]	86.50 [79.80, 91.50]	<0.001
MCH (pg)	29.90 [26.83, 31.58]	30.60 [28.70, 32.40]	28.20 [25.40, 30.70]	<0.001
MCHC (g/L)	327.00 [318.00, 337.00]	329.00 [320.00, 339.00]	322.00 [313.00, 335.00]	0.006
ALT (U/L)	16.85 [12.05, 22.98]	16.85 [12.00, 22.30]	16.85 [13.00, 23.80]	0.919
AST (U/L)	20.00 [17.00, 24.95]	20.00 [18.00, 25.40]	20.00 [17.00, 24.00]	0.435
ALP (U/L)	79.00 [65.15, 90.68]	79.00 [67.00, 87.00]	79.00 [64.00, 104.00]	0.769
TBIL (μmol/L)	9.60 [7.20, 13.01]	9.70 [7.50, 15.10]	9.60 [6.99, 11.20]	0.037
GGT (U/L)	21.60 [16.00, 32.00]	21.60 [19.00, 34.90]	21.60 [13.00, 30.00]	0.036
SCR (μmol/L)	58.00 [51.20, 67.90]	59.30 [52.00, 75.80]	57.00 [50.10, 61.80]	0.004
BUN (mmol/L)	4.95 [3.97, 6.14]	5.09 [4.21, 6.74]	4.70 [3.69, 5.63]	0.001
eGFR (mL/min/1.73 m^2^)	100.95 [92.08, 109.72]	97.39 [84.00, 105.37]	106.90 [97.93, 113.49]	<0.001
NLR	3.47 [2.15, 5.72]	3.33 [1.96, 5.33]	3.54 [2.33, 6.39]	0.127
PLR	189.34 [120.19, 280.91]	179.07 [100.57, 278.89]	193.06 [132.40, 281.58]	0.094
LMR	2.84 [1.87, 4.38]	2.96 [1.90, 5.07]	2.76 [1.76, 3.84]	0.170
SII	698.12 [381.49, 1,223.16]	558.46 [260.56, 1,014.19]	857.99 [502.73, 1,508.55]	<0.001

Continuous variables are presented as median [IQR], and categorical variables are presented as n (%). *P*-values are descriptive. Anti-anemia treatment refers to treatment before enrollment. Patients receiving additional interventions expected to directly affect HGB during follow-up were excluded from the analysis. ALP, alkaline phosphatase; ALT, alanine aminotransferase; AST, aspartate aminotransferase; BMI, body mass index; BUN, blood urea nitrogen; GGT, gamma-glutamyl transferase; HCT, hematocrit; HGB, hemoglobin; LYMPH, lymphocyte count; MCH, mean corpuscular hemoglobin; MCHC, mean corpuscular hemoglobin concentration; MCV, mean corpuscular volume; MONO, monocyte count; NEUT, neutrophil count; PLT, platelet count; RBC, red blood cell count; SCR, serum creatinine; TBIL, total bilirubin; WBC, white blood cell count.

### 3.3 Feature selection

Anemia etiology was selected by all five methods, and age and eGFR were each selected by four methods. PLT was selected by three methods and did not meet the prespecified threshold. The final model, therefore, included anemia etiology, age, and eGFR (Figure 2).

**FIGURE 2:**
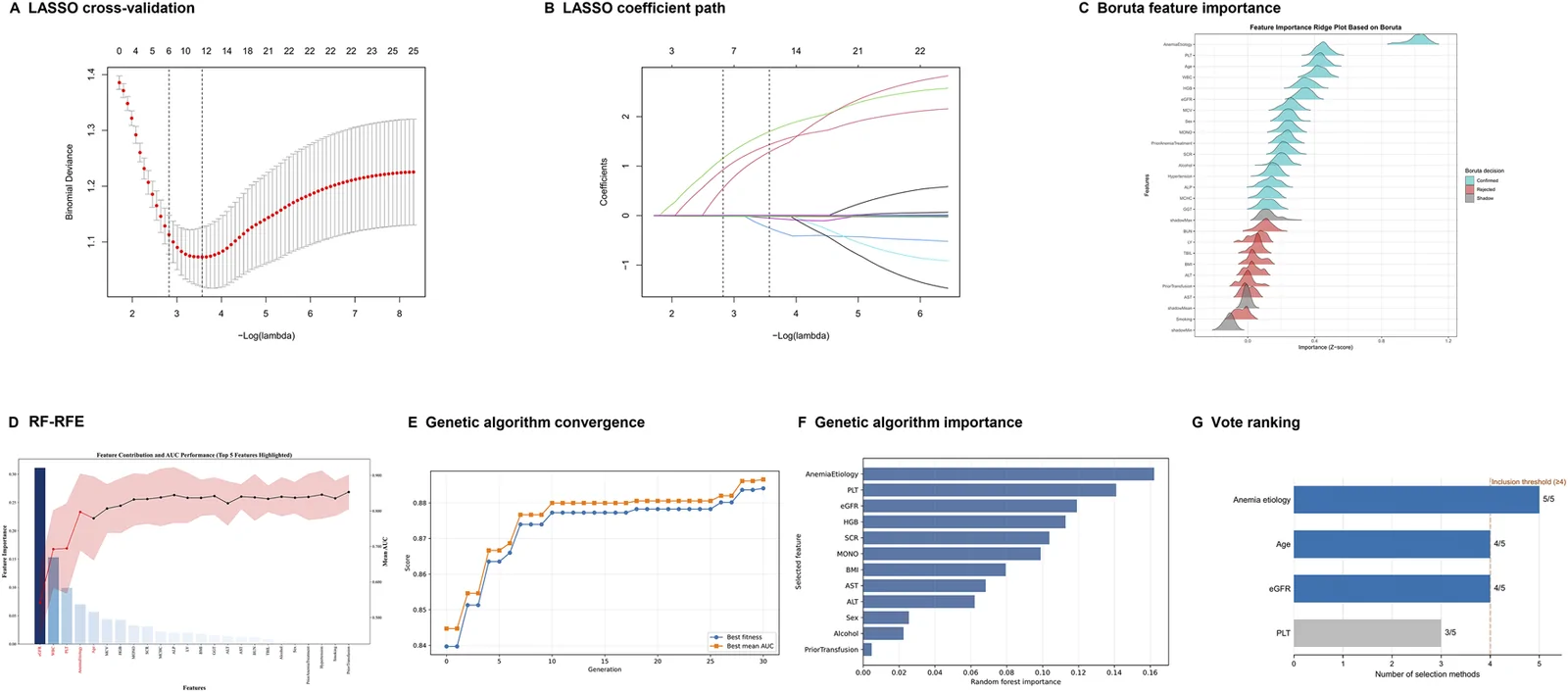
Feature-selection workflow. **(A)** LASSO cross-validation curve. **(B)** LASSO coefficient paths. **(C)** Boruta feature-importance distribution. **(D)** Random-forest recursive feature elimination. **(E)** Genetic-algorithm convergence. **(F)** Genetic-algorithm feature importance. **(G)** Voting across multivariable logistic regression and four machine-learning selection methods. Anemia etiology received 5/5 votes, age and eGFR received 4/5 votes, and PLT received 3/5 votes. The ≥4/5 threshold retained anemia etiology, age, and eGFR.

### 3.4 Model performance

Using the three selected predictors, ET achieved an internal-validation AUC of 0.821 (95% CI, 0.729–0.902) and an external-validation AUC of 0.769 (95% CI, 0.670–0.860) (Figures 3B,C). At a probability threshold of 0.50, sensitivity and specificity in external validation were 0.551 and 0.811.

**FIGURE 3:**
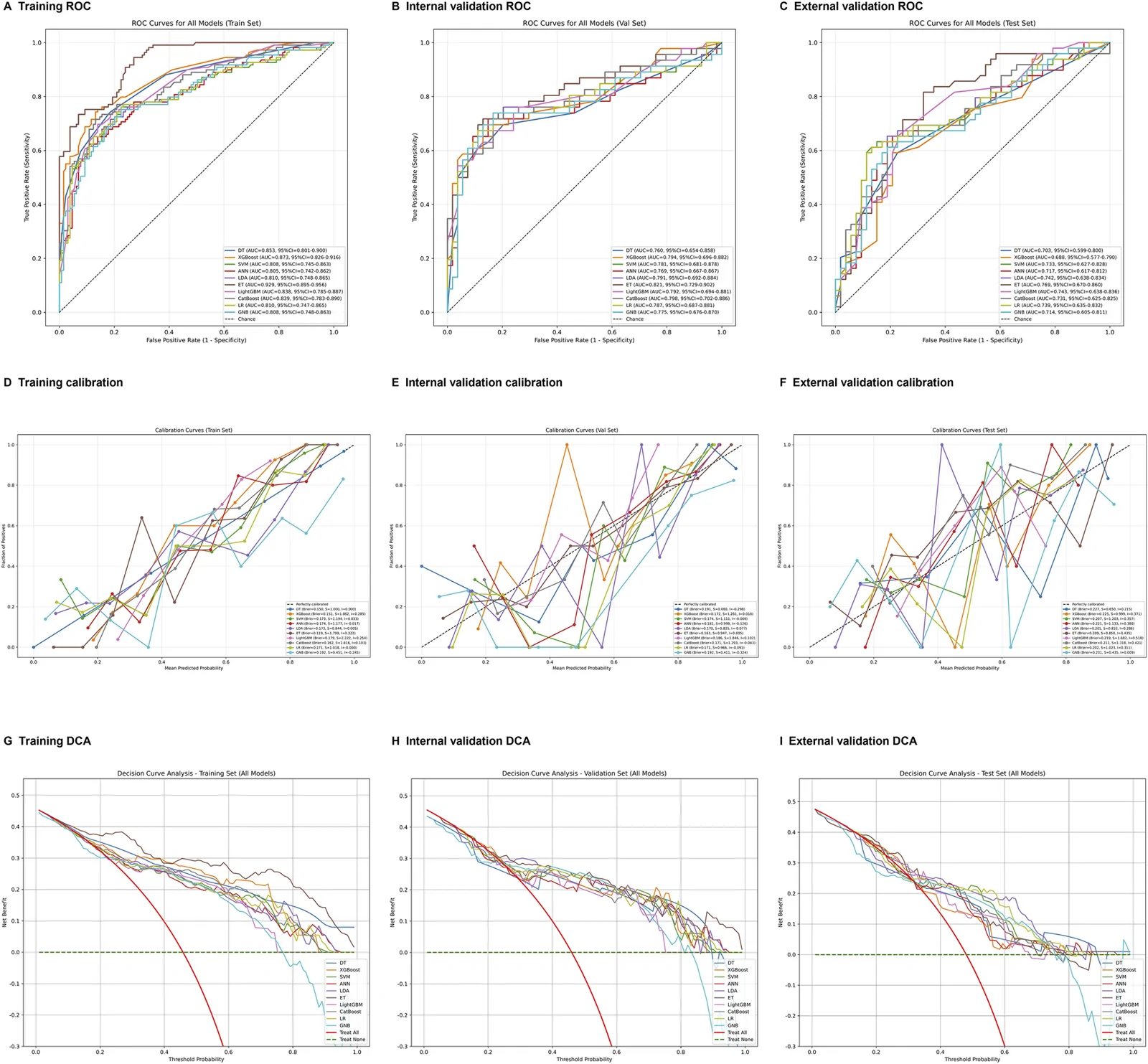
Discrimination, calibration, and decision curve analysis for 10 candidate models. **(A–C)** Receiver operating characteristic curves in the training, internal validation, and external validation cohorts. **(D–F)** Calibration curves in the same cohorts. **(G–I)** Decision curves in the same cohorts.

Calibration was closer to the ideal intercept of 0 and slope of 1 in internal validation than in external validation (Figures 3E,F). Decision curve analyses are shown in Figures 3G–I. Complete discrimination, classification, and calibration estimates for all models and cohorts are reported in Supplementary Table S3.

ET achieved AP values of 0.840 in internal validation and 0.720 in external validation (Figures 4A,B). Figure 4C compares classification metrics across the 10 algorithms in internal validation. Pairwise DeLong tests found no statistically significant AUC differences between ET and the other algorithms; complete *P*-values are provided in Supplementary Figure S4.

**FIGURE 4:**
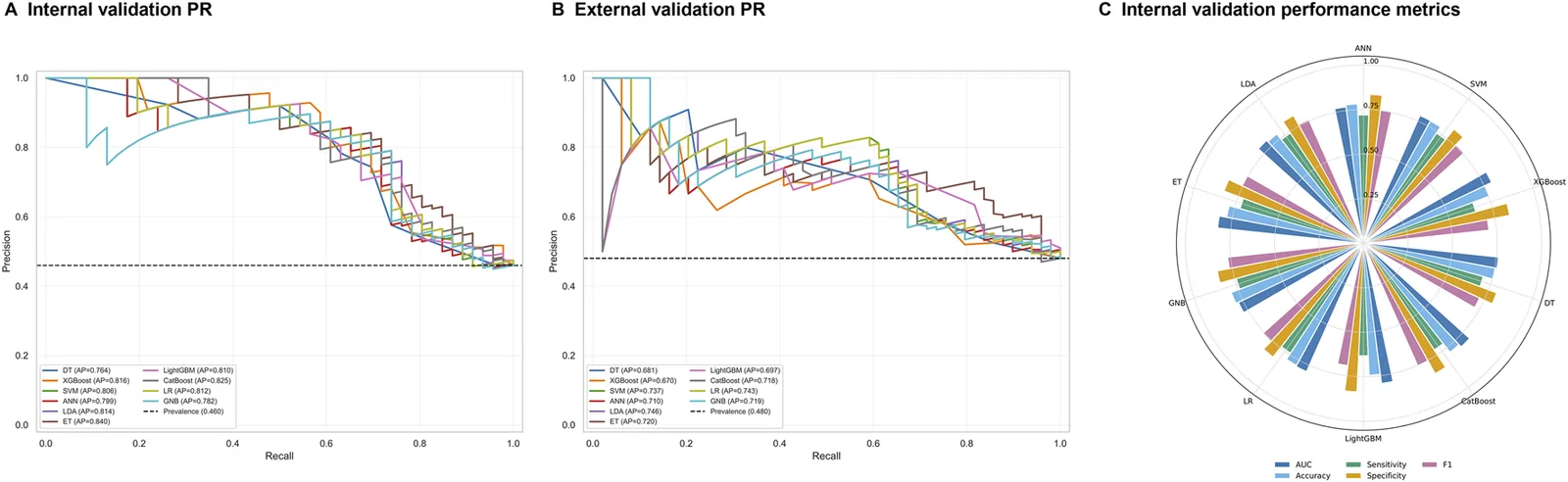
Precision–recall curves and internal-validation performance. **(A)** Precision–recall curves in the internal validation cohort. **(B)** Precision–recall curves in the external validation cohort. **(C)** Polar bar chart comparing the AUC, accuracy, sensitivity, specificity, and F1 score across the 10 algorithms in internal validation.

### 3.5 Model interpretation

Grouped SHAP analysis identified anemia etiology as the largest contributor to the locked ET model’s predictions, followed by age and eGFR (Figures 5A,B). The beeswarm plot showed variation in both the direction and magnitude of contributions across patients. Local SHAP and LIME explanations illustrated contributions to a representative training-set prediction (Figures 5C,D).

**FIGURE 5:**
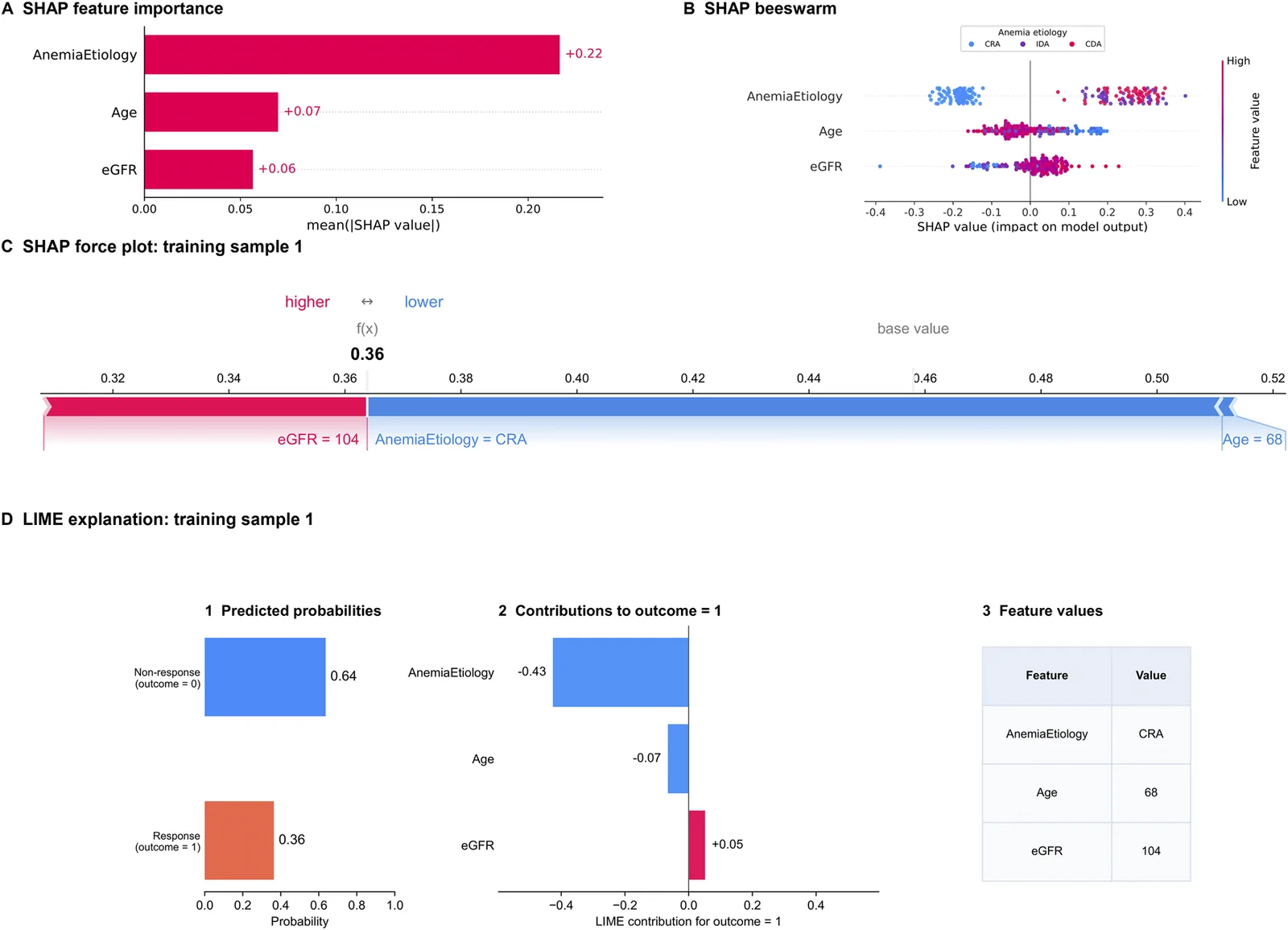
Explanations of the locked ET model’s predictions **(A)** Mean absolute grouped SHAP values for anemia etiology, age, and eGFR. **(B)** Grouped SHAP beeswarm plot. **(C)** Local SHAP force explanation. **(D)** LIME explanation.

SHAP dependence plots showed how contributions to ET predictions varied across the three retained predictors (Figure 6). They showed separation across anemia etiologies and non-linear contribution patterns for age and eGFR.

**FIGURE 6:**
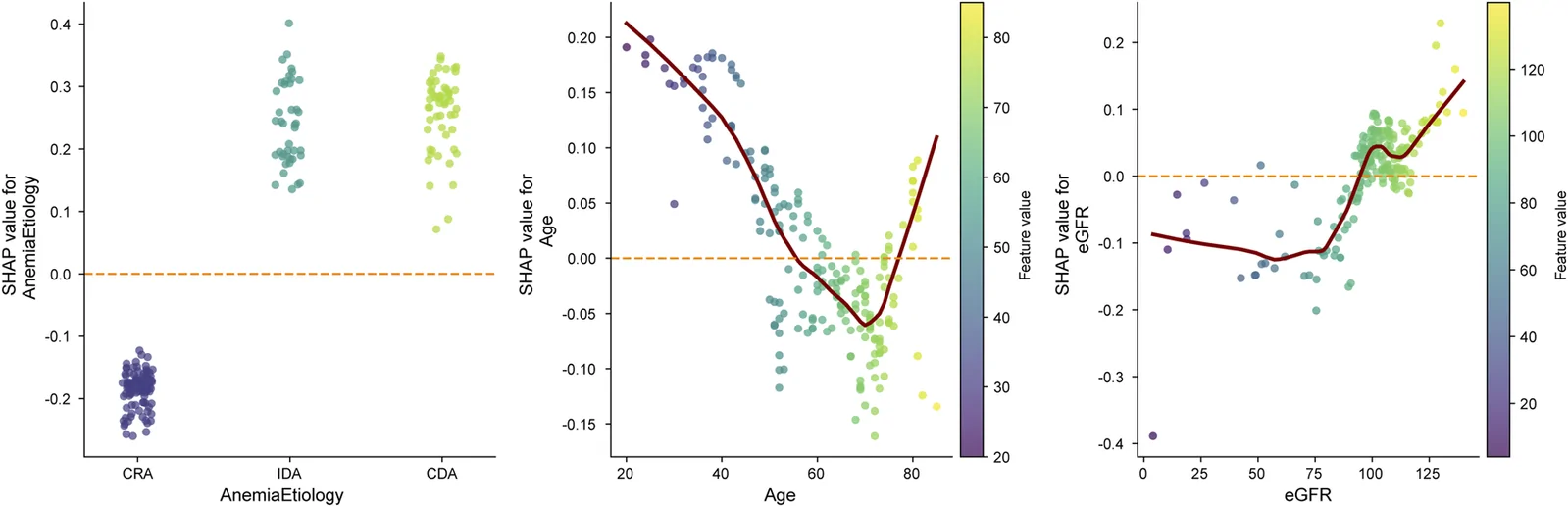
Grouped SHAP dependence plots for the three predictors retained in the ET model: anemia etiology, age, and eGFR.

## 4 Discussion

The main finding of this prospective multicenter study was that an ET model based on anemia etiology, age, and eGFR discriminated between responders and non-responders in four external validation centers. Anemia etiology contributed most strongly to model predictions, while age and eGFR provided additional predictive information. The internal and external validation results support the model’s predictive performance, although differences in discrimination and calibration indicate that further evaluation in larger independent cohorts is warranted.

Previous studies of hemoglobin response have addressed different treatment settings and prediction time-points. In patients with solid tumors receiving erythropoiesis-stimulating therapy, early changes in hemoglobin during treatment provided more predictive information than routine baseline variables (Steinmetz et al., 2007). In women receiving oral iron, baseline hemoglobin, ferritin, hepcidin, and total body iron stores predicted subsequent hemoglobin response (Pei et al., 2021). Machine-learning studies in dialysis populations have incorporated longitudinal hemoglobin, iron-status, and erythropoiesis-stimulating agent data (Barbieri et al., 2015). The present model was restricted to pretreatment variables, allowing response probability to be estimated before measurements from the treatment course become available. This supports assessment at treatment initiation, although it excludes early changes that may provide additional predictive information.

Anemia etiology showed the clearest differences in observed response rates and contributed most strongly to ET predictions. This finding is clinically plausible because cancer-related anemia, iron-deficiency anemia, and anemia of chronic disease involve different pathophysiological mechanisms. A prospective study of 888 patients with solid tumors found associations between hemoglobin and tumor stage, inflammation, iron metabolism, and nutritional status before antineoplastic treatment (Macciò et al., 2015). These findings support the multifactorial nature of cancer-related anemia. Absolute iron deficiency primarily reflects inadequate iron stores. Chronic inflammation can restrict iron availability through hepcidin-mediated mechanisms and impair erythropoiesis despite relatively preserved iron stores (Weiss et al., 2019; Snook et al., 2021). Anemia etiology may summarize differences in inflammation, iron availability, disease burden, nutritional status, and prior treatment exposure. These differences may explain its prominent contribution to the model.

Age and eGFR are closely related to erythropoiesis and anemia. Anemia in older adults is often multifactorial, involving nutritional deficiency, chronic inflammation, declining renal function, reduced erythropoietin activity, and age-related hematopoietic changes (Guralnik et al., 2022; Wacka et al., 2024). Wacka et al. highlighted the combined contributions of low-grade inflammation, renal impairment, and altered erythropoietin responsiveness. Anemia in chronic kidney disease may involve erythropoietin deficiency or resistance, nutritional deficiencies, systemic inflammation, blood loss, and shortened red blood cell survival (Babitt et al., 2026). Higher eGFR and lower inflammatory activity have been associated with a better initial response to darbepoetin alfa in patients with non-dialysis CKD (Hayashi et al., 2021). In our external cohort, neither age nor eGFR clearly separated responders from non-responders, despite contributing to ET predictions. These contributions may involve non-linear relationships with response or interactions with anemia etiology, although the present analyses do not establish such interactions.

The external-validation results provide evidence of predictive performance in patients from independent centers. The ET model achieved an AUC of 0.769, with sensitivity of 0.551 and specificity of 0.811 at the 0.50 threshold. The calibration intercept of 0.435 and slope of 0.850 indicated differences between predicted and observed response probabilities. Guidance emphasizes the evaluation of discrimination, calibration, and clinical utility because patient case mix and clinical setting may affect performance across populations (Riley et al., 2024). Such variation has also been observed in external evaluations of hematologic prediction models (Li et al., 2025b). Our center-based external validation provides encouraging evidence for further evaluation across centers. Larger independent cohorts, with recalibration where needed, may help clarify the model’s generalizability.

The model’s main clinical advantages are its limited number of predictors and their availability before treatment. Anemia etiology, age, and eGFR allow response probability to be estimated without specialized biomarkers or measurements obtained during treatment. The model may support pretreatment response assessment and patient stratification among individuals receiving ZSC.

Several limitations should be considered. First, excluding patients without an evaluable week-8 HGB measurement may have introduced selection bias. Excluding patients receiving additional HGB-affecting interventions may also limit applicability to patients requiring concurrent treatments. Second, anemia etiology was classified using routine clinical diagnoses, although overlapping mechanisms may not be fully represented by a single category. Inflammatory markers, detailed iron indices, and nutritional measures were not uniformly available. Third, the response endpoint depended partly on baseline HGB. Fourth, the external cohort was relatively small, and between-center heterogeneity may have affected the precision of performance and calibration estimates. Finally, all patients received ZSC, and there was no untreated or active-comparator group. The model, therefore, predicts response under observed real-world care and does not estimate the individualized causal treatment effect of ZSC.

## 5 Conclusion

In this prospective multicenter real-world study, 46.4% of patients receiving ZSC achieved an 8-week hemoglobin response. The observed response rates were higher in iron-deficiency anemia and anemia of chronic disease than in cancer-related anemia. An ET model incorporating anemia etiology, age, and eGFR achieved an external-validation AUC of 0.769. Using routinely available baseline information, the model may support individualized assessment of response probability before treatment. Further prospective evaluation is warranted to establish its clinical utility.

## Data Availability

The datasets analyzed in this study are not publicly available because they contain patient-level clinical information from participating centers. De-identified analytic materials may be available from the authors upon reasonable request, subject to institutional and ethical approval. The main analytic outputs are included in the article and Supplementary Material.
