# Supplementary material for: Zaizao Shengxue capsule for anemia: a prospective multicenter real-world study of hemoglobin response and predictive modeling
Source: Front Pharmacol. 2026 Oct 19;17:1957386. doi: 10.3389/fphar.2026.1957386 (PMC13724563; doi:10.3389/fphar.2026.1957386)
Supplement: Supplementary file 1 [file Supplementaryfile1.docx]

**Supplementary material**

This file contains external-validation baseline characteristics; supplementary analyses of missing data, classification, continuous-variable correlations, and internal-validation AUC comparisons; and the botanical and zoological composition of ZSC. It also includes complete performance and tuning results for the ten candidate models, training-cohort out-of-fold ROC curves, participating centers and cohort allocation, VIFs after redundancy screening, and HGB-affecting concomitant treatments.

# Contents

Supplementary Table S1. Baseline characteristics of the external validation cohort	2

Supplementary Figure S1. Missing-data Pareto analysis	3

Supplementary Figure S2. Confusion matrices of all models	4

Supplementary Figure S3. Continuous-variable correlation analysis	5

Supplementary Figure S4. Internal-validation DeLong comparisons	6

Supplementary Table S2. Botanical and zoological composition of Zaizao Shengxue Capsule	7

Supplementary Table S3. Performance of the 10 candidate models in the three study cohorts	8

Supplementary Table S4. Selected hyperparameters and five-fold cross-validation AUCs	9

Supplementary Figure S5. Five-fold cross-validated out-of-fold ROC curves	10

Supplementary Table S5. Participating centers and cohort allocation	11

Supplementary Table S6. Variance inflation factors after redundancy screening	12

Supplementary Table S7. Concomitant treatments potentially affecting HGB during follow-up	13

## Supplementary Table S1. Baseline characteristics of the external validation cohort stratified by 8-week hemoglobin response.

| **Variable** | **Overall (N=102)** | **Nonresponse(N=53)** | **Response (N=49)** | **P** |
| --- | --- | --- | --- | --- |
| Sex |  |  |  | 0.843 |
| Female | 48 (47.06%) | 24 (45.28%) | 24 (48.98%) |  |
| Male | 54 (52.94%) | 29 (54.72%) | 25 (51.02%) |  |
| Anemia etiology |  |  |  | <0.001 |
| Cancer-related anemia | 60 (58.82%) | 43 (81.13%) | 17 (34.69%) |  |
| Iron-deficiency anemia | 27 (26.47%) | 8 (15.09%) | 19 (38.78%) |  |
| Anemia of chronic disease | 15 (14.71%) | 2 (3.77%) | 13 (26.53%) |  |
| Anti-anemia treatment history |  |  |  |  |
| No | 102 (100.00%) | 53 (100.00%) | 49 (100.00%) |  |
| Transfusion history |  |  |  |  |
| No | 102 (100.00%) | 53 (100.00%) | 49 (100.00%) |  |
| Hypertension |  |  |  | 0.027 |
| No | 94 (92.16%) | 52 (98.11%) | 42 (85.71%) |  |
| Yes | 8 (7.84%) | 1 (1.89%) | 7 (14.29%) |  |
| Smoking |  |  |  | 1.000 |
| No | 92 (90.20%) | 48 (90.57%) | 44 (89.80%) |  |
| Yes | 10 (9.80%) | 5 (9.43%) | 5 (10.20%) |  |
| Alcohol drinking |  |  |  | 0.545 |
| No | 90 (88.24%) | 48 (90.57%) | 42 (85.71%) |  |
| Yes | 12 (11.76%) | 5 (9.43%) | 7 (14.29%) |  |
| Age (years) | 64.00 [57.00, 71.00] | 63.00 [57.00, 70.00] | 66.00 [60.00, 73.00] | 0.307 |
| BMI (kg/m²) | 22.48 [19.06, 24.94] | 21.26 [18.71, 24.22] | 22.86 [20.76, 24.97] | 0.105 |
| HGB (g/L) | 96.50 [82.00, 106.75] | 104.00 [96.00, 110.00] | 82.00 [78.00, 96.00] | <0.001 |
| WBC (×10⁹/L) | 5.00 [3.66, 6.62] | 5.00 [3.95, 6.57] | 5.00 [3.50, 6.67] | 0.928 |
| RBC (×10¹²/L) | 3.44 [3.05, 3.82] | 3.52 [3.25, 3.88] | 3.29 [2.95, 3.67] | 0.024 |
| PLT (×10⁹/L) | 203.50 [120.50, 264.75] | 185.00 [125.00, 264.00] | 216.00 [116.00, 268.00] | 0.812 |
| LYMPH (×10⁹/L) | 1.06 [0.82, 1.42] | 1.06 [0.93, 1.46] | 1.06 [0.73, 1.23] | 0.104 |
| MONO (×10⁹/L) | 0.40 [0.28, 0.48] | 0.40 [0.30, 0.47] | 0.40 [0.28, 0.48] | 0.848 |
| NEUT (×10⁹/L) | 3.70 [2.69, 4.50] | 3.70 [2.87, 4.51] | 3.70 [2.55, 4.46] | 0.981 |
| HCT (%) | 31.70 [28.15, 35.00] | 33.00 [31.00, 37.00] | 28.80 [25.80, 32.20] | <0.001 |
| MCV (fL) | 88.65 [79.00, 95.03] | 91.20 [85.00, 96.40] | 85.30 [76.80, 92.10] | 0.016 |
| MCH (pg) | 28.90 [25.38, 31.20] | 29.50 [27.80, 31.90] | 27.70 [23.30, 30.40] | 0.050 |
| MCHC (g/L) | 318.00 [305.25, 329.00] | 320.00 [310.00, 329.00] | 313.00 [303.00, 329.00] | 0.152 |
| ALT (U/L) | 20.00 [16.21, 25.00] | 20.00 [16.85, 29.00] | 20.00 [16.00, 23.80] | 0.578 |
| AST (U/L) | 22.00 [20.00, 28.50] | 22.00 [20.00, 29.00] | 22.00 [20.00, 27.00] | 0.699 |
| ALP (U/L) | 79.60 [65.12, 101.50] | 79.00 [66.70, 100.00] | 79.80 [64.00, 117.00] | 0.904 |
| TBIL (μmol/L) | 9.92 [7.91, 14.00] | 9.60 [8.30, 13.32] | 10.00 [7.10, 14.00] | 0.867 |
| GGT (U/L) | 22.85 [21.60, 38.58] | 23.40 [21.60, 46.00] | 22.50 [20.00, 36.00] | 0.317 |
| SCR (μmol/L) | 63.20 [57.25, 75.75] | 66.50 [58.00, 78.00] | 63.00 [52.60, 69.50] | 0.067 |
| BUN (mmol/L) | 4.95 [4.20, 6.40] | 5.19 [4.36, 6.40] | 4.85 [3.60, 6.40] | 0.237 |
| eGFR | 98.11 [85.69, 104.03] | 98.07 [84.23, 104.12] | 98.38 [88.74, 103.78] | 0.627 |
| NLR | 3.40 [2.13, 4.53] | 3.47 [2.01, 3.61] | 3.33 [2.33, 5.24] | 0.226 |
| PLR | 175.05 [109.18, 263.47] | 155.56 [111.74, 227.23] | 200.90 [108.33, 270.42] | 0.128 |
| LMR | 2.79 [2.40, 4.26] | 2.84 [2.60, 4.51] | 2.71 [2.15, 4.03] | 0.392 |
| SII | 619.50 [312.19, 979.72] | 512.07 [339.18, 913.71] | 793.98 [280.76, 1247.52] | 0.241 |

Note. Continuous variables are presented as median [IQR] and categorical variables as n (%). P values are descriptive. Anti-anemia treatment refers to treatment before enrollment. Patients receiving additional interventions expected to directly affect HGB during follow-up were excluded from the analysis. Abbreviations are defined in the main manuscript.

## Supplementary Figure S1. Missing-data Pareto analysis

Supplementary Figure S1. Missing-data Pareto plots before imputation in the development cohort (A; n=338) and external validation cohort (B; n=102). Bars represent the percentage of missing values for each variable. The line represents the cumulative share of all missing values.

## Supplementary Figure S2. Confusion matrices of all models

Supplementary Figure S2. Confusion matrices for the ten candidate algorithms in the training (n=238), internal validation (n=100), and external validation (n=102) cohorts. Rows represent observed outcomes and columns represent predicted outcomes at a probability threshold of 0.50. Class 0 denotes nonresponse and class 1 denotes response.

## Supplementary Figure S3. Continuous-variable correlation analysis

Supplementary Figure S3. Pearson and Spearman correlation coefficients for continuous variables in the training cohort (n=238). Pearson coefficients are displayed below the diagonal and Spearman coefficients above it. Strong correlations were defined as |r| ≥0.80 with FDR-adjusted P<0.05. Redundant variables were reviewed before feature selection, considering clinical interpretability and VIFs.

## Supplementary Figure S4. Internal-validation DeLong comparisons

Supplementary Figure S4. Heatmap of pairwise DeLong P values comparing model AUCs in the internal validation cohort (n=100).

## Supplementary Table S2. Botanical and zoological composition of Zaizao Shengxue Capsule

| **Chinese name** | **Species (authority) [family; pharmacopoeial drug name]** | **Part used** | **Processing/form** |
| --- | --- | --- | --- |
| Tu Si Zi | Cuscuta chinensis Lam. [Convolvulaceae; Cuscutae Semen] | Ripe seed | Wine-processed (as labeled) |
| Hong Shen | Panax ginseng C.A.Mey. [Araliaceae; Ginseng Radix et Rhizoma Rubra] | Steamed and dried root | Root crown/rhizome removed (as labeled) |
| E Jiao | *Equus asinus* L. (domestic donkey) [Equidae; Asini Corii Colla] | Hide-derived gelatin | Processed animal-derived pharmacopoeial drug |
| Dang Gui | Angelica sinensis (Oliv.) Diels [Apiaceae; Angelicae Sinensis Radix] | Root | Dried, raw |
| Ji Xue Teng | Spatholobus suberectus Dunn [Fabaceae; Spatholobi Caulis] | Stem | Dried, raw |
| Nu Zhen Zi | Ligustrum lucidum W.T.Aiton [Oleaceae; Ligustri Lucidi Fructus] | Ripe fruit | Dried, raw |
| Huang Qi | Astragalus mongholicus Bunge (pharmacopoeial synonyms include Astragalus membranaceus (Fisch.) Bunge and A. membranaceus var. mongholicus (Bunge) P.K.Hsiao) [Fabaceae; Astragali Radix] | Root | Dried, raw |
| Bai Shao | Paeonia lactiflora Pall. [Paeoniaceae; Paeoniae Radix Alba] | Root | Peeled, briefly boiled, and dried |
| Yi Mu Cao | Leonurus japonicus Houtt. [Lamiaceae; Leonuri Herba] | Aerial part | Dried, raw |
| Shu Di Huang | Rehmannia glutinosa (Gaertn.) Libosch. ex DC. [Orobanchaceae; Rehmanniae Radix Praeparata] | Tuberous root | Prepared/steamed |
| Zhi He Shou Wu | Pleuropterus multiflorus (Thunb.) Turcz. ex Nakai (syn. Polygonum multiflorum Thunb.) [Polygonaceae; Polygoni Multiflori Radix Praeparata] | Tuberous root | Prepared with black-soybean decoction |
| Jiu Huang Jing | Polygonatum sibiricum Redouté, P. cyrtonema Hua, or P. kingianum Collett & Hemsl. [Asparagaceae; Polygonati Rhizoma] | Rhizome | Wine-processed |
| Yin Yang Huo | Epimedium brevicornu Maxim., E. sagittatum (Siebold & Zucc.) Maxim., E. pubescens Maxim., or E. koreanum Nakai [Berberidaceae; Epimedii Folium] | Leaf | Dried, raw |
| Lu Rong | Cervus nippon Temminck or Cervus elaphus Linnaeus [Cervidae; Cervi Cornu Pantotrichum] | Unossified pilose antler | Dehaired (as labeled) |
| Mai Dong | Ophiopogon japonicus (Thunb.) Ker Gawl. [Asparagaceae; Ophiopogonis Radix] | Tuberous root | Dried, raw |
| Dang Shen | Codonopsis pilosula (Franch.) Nannf., C. pilosula subsp. handeliana (Nannf.) D.Y.Hong & L.M.Ma (syn. C. pilosula var. modesta), or C. pilosula subsp. tangshen (Oliv.) D.Y.Hong (syn. C. tangshen Oliv.) [Campanulaceae; Codonopsis Radix] | Root | Dried, raw |
| Xian He Cao | Agrimonia pilosa Ledeb. [Rosaceae; Agrimoniae Herba] | Aerial part | Dried, raw |
| Fu Chao Bai Zhu | Atractylodes macrocephala Koidz. [Asteraceae; Atractylodis Macrocephalae Rhizoma] | Rhizome | Bran-fried |
| Gou Qi Zi | Lycium barbarum L. [Solanaceae; Lycii Fructus] | Ripe fruit | Dried, raw |
| Yan Bu Gu Zhi | Cullen corylifolium (L.) Medik. (syn. Psoralea corylifolia L.) [Fabaceae; Psoraleae Fructus] | Ripe fruit | Salt-processed |
| Mo Han Lian | Eclipta prostrata (L.) L. [Asteraceae; Ecliptae Herba] | Aerial part | Dried, raw |

Note. The study used finished-product batch 20230504, identified as batch No. 230504 in the manufacturer's inspection report, which is provided as a separate Supplementary File. Ingredient order and labeled processing forms follow the verified product information. Pharmacopoeial drug names and source taxa follow the Pharmacopoeia of the People's Republic of China (2020 edition). For ethical sourcing and traceability, Asini Corii Colla (E Jiao) was procured through documented, regulated pharmaceutical channels from Guilu Pharmaceutical Group Co., Ltd., whose Asini Corii Colla product is approved by the National Medical Products Administration (NMPA; approval No. Z41020163). The supplied Asini Corii Colla is subject to quality control and testing in accordance with the Chinese Pharmacopoeia. The investigators did not acquire or process donkey hides or conduct live-animal procedures.

## Supplementary Table S3. Performance of the 10 candidate models in the training, internal-validation, and external-validation cohorts

| **Model** | **Cohort** | **AUC (95% CI)** | **AP** | **Accuracy** | **Sensitivity** | **Specificity** | **Precision** | **NPV** | **F1** | **Brier** | **Calibration intercept** | **Calibration slope** |
| --- | --- | --- | --- | --- | --- | --- | --- | --- | --- | --- | --- | --- |
| DT | Training | 0.853 (0.801-0.900) | 0.831 | 0.782 | 0.743 | 0.814 | 0.771 | 0.789 | 0.757 | 0.150 | 0.000 | 1.000 |
| DT | Internal validation | 0.760 (0.654-0.858) | 0.764 | 0.750 | 0.696 | 0.796 | 0.744 | 0.754 | 0.719 | 0.191 | -0.298 | 0.060 |
| DT | External validation | 0.703 (0.599-0.800) | 0.681 | 0.686 | 0.592 | 0.774 | 0.707 | 0.672 | 0.644 | 0.227 | 0.215 | 0.650 |
| XGBoost | Training | 0.873 (0.827-0.916) | 0.872 | 0.807 | 0.716 | 0.884 | 0.839 | 0.786 | 0.772 | 0.151 | 0.285 | 1.862 |
| XGBoost | Internal validation | 0.794 (0.696-0.882) | 0.816 | 0.750 | 0.652 | 0.833 | 0.769 | 0.738 | 0.706 | 0.172 | 0.018 | 1.261 |
| XGBoost | External validation | 0.688 (0.577-0.790) | 0.670 | 0.647 | 0.469 | 0.811 | 0.697 | 0.623 | 0.561 | 0.225 | 0.371 | 0.999 |
| SVM | Training | 0.808 (0.745-0.863) | 0.816 | 0.752 | 0.697 | 0.798 | 0.745 | 0.757 | 0.720 | 0.173 | 0.033 | 1.194 |
| SVM | Internal validation | 0.781 (0.681-0.878) | 0.806 | 0.780 | 0.739 | 0.815 | 0.773 | 0.786 | 0.756 | 0.174 | -0.069 | 1.111 |
| SVM | External validation | 0.733 (0.627-0.828) | 0.737 | 0.745 | 0.612 | 0.868 | 0.811 | 0.708 | 0.698 | 0.207 | 0.357 | 1.203 |
| ANN | Training | 0.805 (0.742-0.862) | 0.809 | 0.752 | 0.688 | 0.806 | 0.750 | 0.754 | 0.718 | 0.174 | -0.017 | 1.177 |
| ANN | Internal validation | 0.769 (0.667-0.867) | 0.799 | 0.780 | 0.717 | 0.833 | 0.786 | 0.776 | 0.750 | 0.181 | -0.126 | 0.999 |
| ANN | External validation | 0.717 (0.617-0.812) | 0.710 | 0.657 | 0.429 | 0.868 | 0.750 | 0.622 | 0.545 | 0.221 | 0.380 | 1.133 |
| LDA | Training | 0.810 (0.748-0.865) | 0.816 | 0.761 | 0.688 | 0.822 | 0.765 | 0.757 | 0.725 | 0.173 | 0.005 | 0.844 |
| LDA | Internal validation | 0.791 (0.692-0.884) | 0.814 | 0.780 | 0.739 | 0.815 | 0.773 | 0.786 | 0.756 | 0.170 | -0.077 | 0.825 |
| LDA | External validation | 0.742 (0.638-0.834) | 0.746 | 0.725 | 0.633 | 0.811 | 0.756 | 0.705 | 0.689 | 0.201 | 0.286 | 0.832 |
| ET | Training | 0.929 (0.895-0.956) | 0.925 | 0.832 | 0.734 | 0.915 | 0.879 | 0.803 | 0.800 | 0.119 | 0.322 | 1.799 |
| ET | Internal validation | 0.821 (0.729-0.902) | 0.840 | 0.780 | 0.717 | 0.833 | 0.786 | 0.776 | 0.750 | 0.161 | 0.005 | 0.947 |
| ET | External validation | 0.769 (0.670-0.860) | 0.720 | 0.686 | 0.551 | 0.811 | 0.730 | 0.662 | 0.628 | 0.209 | 0.435 | 0.850 |
| LightGBM | Training | 0.838 (0.785-0.887) | 0.816 | 0.773 | 0.633 | 0.891 | 0.831 | 0.742 | 0.719 | 0.179 | 0.254 | 2.222 |
| LightGBM | Internal validation | 0.792 (0.694-0.881) | 0.810 | 0.740 | 0.630 | 0.833 | 0.763 | 0.726 | 0.690 | 0.186 | 0.102 | 1.846 |
| LightGBM | External validation | 0.743 (0.638-0.836) | 0.697 | 0.657 | 0.388 | 0.906 | 0.792 | 0.615 | 0.521 | 0.219 | 0.518 | 1.682 |
| CatBoost | Training | 0.839 (0.783-0.890) | 0.842 | 0.794 | 0.706 | 0.868 | 0.819 | 0.778 | 0.759 | 0.162 | 0.103 | 1.616 |
| CatBoost | Internal validation | 0.798 (0.702-0.886) | 0.825 | 0.780 | 0.717 | 0.833 | 0.786 | 0.776 | 0.750 | 0.171 | -0.043 | 1.293 |
| CatBoost | External validation | 0.731 (0.625-0.825) | 0.718 | 0.667 | 0.510 | 0.811 | 0.714 | 0.642 | 0.595 | 0.211 | 0.421 | 1.318 |
| LR | Training | 0.810 (0.747-0.865) | 0.817 | 0.765 | 0.697 | 0.822 | 0.768 | 0.763 | 0.731 | 0.171 | -0.000 | 1.018 |
| LR | Internal validation | 0.787 (0.687-0.881) | 0.812 | 0.780 | 0.739 | 0.815 | 0.773 | 0.786 | 0.756 | 0.171 | -0.091 | 0.966 |
| LR | External validation | 0.739 (0.635-0.832) | 0.743 | 0.735 | 0.633 | 0.830 | 0.775 | 0.710 | 0.697 | 0.202 | 0.311 | 1.023 |
| GNB | Training | 0.808 (0.748-0.863) | 0.806 | 0.761 | 0.725 | 0.791 | 0.745 | 0.773 | 0.735 | 0.192 | -0.245 | 0.451 |
| GNB | Internal validation | 0.775 (0.675-0.870) | 0.782 | 0.790 | 0.739 | 0.833 | 0.791 | 0.789 | 0.764 | 0.192 | -0.324 | 0.411 |
| GNB | External validation | 0.714 (0.605-0.811) | 0.719 | 0.706 | 0.633 | 0.774 | 0.721 | 0.695 | 0.674 | 0.231 | 0.009 | 0.435 |

**Note.** Average precision (AP), Brier scores, calibration intercepts, and calibration slopes were recomputed from saved probabilities generated by the locked models. Classification metrics were calculated at a probability threshold of 0.50. Training estimates represent apparent performance. No external-validation observations were used for feature selection, hyperparameter tuning, or final algorithm selection. AUC, area under the receiver operating characteristic curve; AP, average precision; CI, confidence interval; NPV, negative predictive value.

## Supplementary Table S4. Selected hyperparameters and stratified five-fold cross-validation AUCs for the 10 candidate models

| **Model** | **Best mean CV AUC** | **Selected hyperparameters** |
| --- | --- | --- |
| ANN | 0.809 | activation=relu; hidden_layer_sizes=(100,) |
| LR | 0.807 | C=10; class_weight=None; penalty=l2 |
| LDA | 0.805 | n_components=None; shrinkage=None; solver=svd |
| ET | 0.804 | max_depth=10; max_features=sqrt; min_samples_leaf=1; min_samples_split=10; n_estimators=300 |
| CatBoost | 0.800 | depth=3; iterations=200; l2_leaf_reg=5; learning_rate=0.01 |
| SVM | 0.789 | C=0.1; degree=2; gamma=scale; kernel=linear |
| GNB | 0.786 | var_smoothing=1e-12 |
| XGBoost | 0.761 | learning_rate=0.01; max_depth=3; n_estimators=200; subsample=1.0 |
| DT | 0.742 | ccp_alpha=0.0; max_depth=5; max_features=sqrt; min_samples_split=20 |
| LightGBM | 0.728 | colsample_bytree=1.0; learning_rate=0.01; max_depth=3; n_estimators=100; num_leaves=15; subsample=0.8 |

**Note.** Hyperparameters were selected by stratified five-fold cross-validation in the training cohort (n=238), with mean AUC as the optimization criterion. Only selected parameters are shown; unlisted settings retain the values specified in the locked analysis script. ANN, artificial neural network; CV, cross-validation; DT, decision tree; ET, extremely randomized trees (Extra Trees); GNB, Gaussian naive Bayes; LDA, linear discriminant analysis; LR, logistic regression; SVM, support vector machine.

## Supplementary Figure S5. Five-fold cross-validated out-of-fold ROC curves

**Supplementary** Figure S5. Pooled out-of-fold ROC curves for the ten candidate algorithms in the training cohort (n=238). Each curve is based on predictions from stratified five-fold cross-validation. The diagonal dashed line represents chance discrimination. AUC labels are pooled out-of-fold estimates and differ from the mean fold AUCs used for hyperparameter selection in Supplementary Table S4.

## Supplementary Table S5. Participating centers and cohort allocation

| **Center ID** | **Participating center and department** | **Cohort allocation** |
| --- | --- | --- |
| 1 | Dongzhimen Hospital, Beijing University of Chinese Medicine - Hematology and Oncology | Development |
| 2 | Guang'an District People's Hospital, Guang'an, Sichuan - Oncology | Development |
| 3 | Guang'an District People's Hospital, Guang'an, Sichuan - Obstetrics and Gynecology | Development |
| 4 | Guang'an District People's Hospital, Guang'an, Sichuan - Neurology | External validation |
| 5 | First Affiliated Hospital of Henan University of Science and Technology - Internal Medicine | Development |
| 6 | Second Affiliated Hospital of Henan University of Science and Technology - Medical Oncology | Development |
| 7 | West China Hospital, Sichuan University - Oncology | External validation |
| 8 | Suzhou Hospital of Integrated Chinese Medicine and Biomedicine - Medical Oncology | Development |
| 9 | Qinhuangdao Third Hospital - Oncology | External validation |
| 10 | Kunshan Hospital of Chinese Medicine - Nephrology | Development |
| 11 | Linzhou People's Hospital, Anyang - Oncology | Development |
| 12 | Dezhou Second People's Hospital - Thoracic Surgery | External validation |
| 13 | Mianyang Third People's Hospital - Hematology | Development |
| 14 | Second Affiliated Hospital of Guizhou Medical University - Oncology | Development |

Note. Centers 4, 7, 9, and 12 formed the external validation cohort; the remaining 10 centers formed the development cohort.

## Supplementary Table S6. Variance inflation factors after redundancy screening in the training cohort

| **Variable** | **VIF** | **Interpretation** |
| --- | --- | --- |
| eGFR | 3.966 | Acceptable (<5) |
| PriorAnemiaTreatment | 3.534 | Acceptable (<5) |
| SCR | 3.352 | Acceptable (<5) |
| PriorTransfusion | 3.175 | Acceptable (<5) |
| AST | 2.818 | Acceptable (<5) |
| ALT | 2.559 | Acceptable (<5) |
| Age | 2.496 | Acceptable (<5) |
| Sex | 2.185 | Acceptable (<5) |
| MONO | 2.115 | Acceptable (<5) |
| WBC | 2.106 | Acceptable (<5) |
| GGT | 2.068 | Acceptable (<5) |
| BUN | 1.930 | Acceptable (<5) |
| AnemiaEtiology_2_vs_1 | 1.784 | Acceptable (<5) |
| MCHC | 1.737 | Acceptable (<5) |
| ALP | 1.730 | Acceptable (<5) |
| Smoking | 1.718 | Acceptable (<5) |
| MCV | 1.700 | Acceptable (<5) |
| AnemiaEtiology_3_vs_1 | 1.672 | Acceptable (<5) |
| Alcohol | 1.646 | Acceptable (<5) |
| Hypertension | 1.424 | Acceptable (<5) |
| LY | 1.390 | Acceptable (<5) |
| PLT | 1.386 | Acceptable (<5) |
| HGB | 1.348 | Acceptable (<5) |
| TBIL | 1.277 | Acceptable (<5) |
| BMI | 1.207 | Acceptable (<5) |

Note. VIFs were calculated in the training cohort (n=238) after correlation and redundancy screening. Anemia etiology was represented by two indicator variables, with cancer-related anemia (category 1) as the reference. VIF >10 indicated marked multicollinearity, VIF 5–10 warranted review, and VIF <5 was considered acceptable. The maximum final VIF was 3.966.

## Supplementary Table S7. Concomitant treatments potentially affecting HGB during follow-up

| Category of concomitant treatment potentially affecting HGB | Patients, n | Main treatments included |
| --- | --- | --- |
| Antitumor therapy | 40 | Chemotherapy, targeted therapy, immunotherapy, etc. |
| Iron supplementation and other anemia-directed therapy | 32 | Iron preparations, folic acid, vitamin B12, etc. |
| Blood transfusion and erythropoiesis-directed therapy | 10 | Red blood cell transfusion/blood products, erythropoietin (EPO), roxadustat, etc. |
| Total | 82 | — |

Of 589 enrolled patients, 77 lacked an evaluable week-8 HGB outcome. Concomitant treatments were reviewed throughout the 8-week follow-up period. Eighty-two patients received interventions considered likely to directly affect HGB, including antitumor therapy, iron supplementation or other anemia-directed therapy, blood transfusion, and erythropoiesis-directed therapy (Supplementary Table S7). Ten of these patients also lacked an evaluable outcome. Excluding the 77 patients with missing outcomes and an additional 72 patients receiving such interventions left 440 patients in the final prediction-model analysis.
